# Supplementary material for: Smooth Interpolating Curves with Local Control and Monotone Alternating Curvature
Source: Comput Graph Forum. 2022 Oct 6;41(5):25–38. doi: 10.1111/cgf.14600 (PMC9827861; doi:10.1111/cgf.14600)
Supplement: Supplementary file 1 — Supplement Material [file CGF-41-25-s001.zip › Local-Smooth-Interpolating-MonoCurvature/extern/clothoids/submodules/Utils/docs/api-cpp/program_listing_file_Utils_Quaternion.hxx.html]

Program Listing for File Quaternion.hxx — Utils v1.2

### Navigation

- index
- toc
- Utils »
- Program Listing for File Quaternion.hxx

# Program Listing for File Quaternion.hxx¶

↰ Return to documentation for file (`Utils/Quaternion.hxx`)

```
/*--------------------------------------------------------------------------*\
 |                                                                          |
 |  Copyright (C) 2011                                                      |
 |                                                                          |
 |         , __                 , __                                        |
 |        /|/  \               /|/  \                                       |
 |         | __/ _   ,_         | __/ _   ,_                                |
 |         |   \|/  /  |  |   | |   \|/  /  |  |   |                        |
 |         |(__/|__/   |_/ \_/|/|(__/|__/   |_/ \_/|/                       |
 |                           /|                   /|                        |
 |                           \|                   \|                        |
 |                                                                          |
 |      Enrico Bertolazzi                                                   |
 |      Dipartimento di Ingegneria Industriale                              |
 |      Universita` degli Studi di Trento                                   |
 |      email: enrico.bertolazzi@unitn.it                                   |
 |                                                                          |
\*--------------------------------------------------------------------------*/


#pragma once

#ifndef DOXYGEN_SHOULD_SKIP_THIS
#ifndef UTILS_QUATERNION_dot_HH
#define UTILS_QUATERNION_dot_HH
#endif

namespace Utils {

  #ifndef DOXYGEN_SHOULD_SKIP_THIS
  using std::ostream;
  #endif

  template <typename T>
  class Quaternion {
  public:
    typedef T real_type;

  private:
    real_type m_Q[4];

  public:

    Quaternion() {
      m_Q[0] = m_Q[1] = m_Q[2] = m_Q[3] = 0;
    }

    Quaternion(
      real_type A,
      real_type B,
      real_type C,
      real_type D
    ) {
      m_Q[0] = A;
      m_Q[1] = B;
      m_Q[2] = C;
      m_Q[3] = D;
    }

    void
    setup(
      real_type A,
      real_type B,
      real_type C,
      real_type D
    ) {
      m_Q[0] = A;
      m_Q[1] = B;
      m_Q[2] = C;
      m_Q[3] = D;
    }

    void
    print( ostream & os) const {
      os << "[ "
         << m_Q[0] << ", "
         << m_Q[1] << "i, "
         << m_Q[2] << "j, "
         << m_Q[3] << "k ]";
    }

    real_type
    operator [] (int i) const { return m_Q[i]; }

    void
    conj() { m_Q[1] = -m_Q[1]; m_Q[2] = -m_Q[2]; m_Q[3] = -m_Q[3]; }

    void
    invert() {
      real_type bf = 1/(m_Q[0]*m_Q[0] + m_Q[1]*m_Q[1] + m_Q[2]*m_Q[2] + m_Q[3]*m_Q[3]);
      m_Q[0] *= bf; bf = -bf; m_Q[1] *= bf; m_Q[2] *= bf; m_Q[3] *= bf;
    }

    real_type
    norm() const {
      return sqrt(m_Q[0]*m_Q[0] + m_Q[1]*m_Q[1] + m_Q[2]*m_Q[2] + m_Q[3]*m_Q[3]);
    }

    void
    rotate( real_type const v[3], real_type w[3] ) const {
      w[0] = ( m_Q[0] * m_Q[0] + m_Q[1] * m_Q[1] ) * v[0]
           + ( m_Q[1] * m_Q[2] - m_Q[0] * m_Q[3] ) * v[1]
           + ( m_Q[1] * m_Q[3] + m_Q[0] * m_Q[2] ) * v[2];

      w[1] = ( m_Q[1] * m_Q[2] + m_Q[0] * m_Q[3] ) * v[0]
           + ( m_Q[0] * m_Q[0] + m_Q[2] * m_Q[2] ) * v[1]
           + ( m_Q[2] * m_Q[3] - m_Q[0] * m_Q[1] ) * v[2];

      w[2] = ( m_Q[1] * m_Q[3] - m_Q[0] * m_Q[2] ) * v[0]
           + ( m_Q[2] * m_Q[3] + m_Q[0] * m_Q[1] ) * v[1]
           + ( m_Q[0] * m_Q[0] + m_Q[3] * m_Q[3] ) * v[2];

      w[0] = 2*w[0] - v[0];
      w[1] = 2*w[1] - v[1];
      w[2] = 2*w[2] - v[2];
    }

    real_type
    toAxis( real_type axis[3] ) const {
      real_type sin_phi = sqrt( m_Q[1]*m_Q[1] + m_Q[2]*m_Q[2] + m_Q[3]*m_Q[3] );
      real_type cos_phi = m_Q[0];
      real_type angle   = 2 * atan2( sin_phi, cos_phi );
      if ( sin_phi == 0 ) {
        axis[0] = 1; axis[1] = axis[2] = 0;
      } else {
        axis[0] = m_Q[1] / sin_phi;
        axis[1] = m_Q[2] / sin_phi;
        axis[2] = m_Q[3] / sin_phi;
      }
      return angle;
    }

    void
    toMatrix( real_type mat[3][3] ) const {
      real_type axis[3];
      real_type angle = toAxis( axis );
      real_type ca    = cos( angle );
      real_type sa    = sin( angle );

      mat[0][0] =              axis[0] * axis[0] + ca * ( 1 - axis[0] * axis[0] );
      mat[1][0] = ( 1 - ca ) * axis[0] * axis[1] - sa * axis[2];
      mat[2][0] = ( 1 - ca ) * axis[0] * axis[2] + sa * axis[1];

      mat[0][1] = ( 1 - ca ) * axis[1] * axis[0] + sa * axis[2];
      mat[1][1] =              axis[1] * axis[1] + ca * ( 1 - axis[1] * axis[1] );
      mat[2][1] = ( 1 - ca ) * axis[1] * axis[2] - sa * axis[0];

      mat[0][2] = ( 1 - ca ) * axis[2] * axis[0] - sa * axis[1];
      mat[1][2] = ( 1 - ca ) * axis[2] * axis[1] + sa * axis[0];
      mat[2][2] =              axis[2] * axis[2] + ca * ( 1 - axis[2] * axis[2] );
    }

  };

  template <typename T>
  inline
  Quaternion<T>
  operator * ( Quaternion<T> const & a, Quaternion<T> const & b ) {
    return Quaternion<T>( a[0] * b[0] - a[1] * b[1] - a[2] * b[2] - a[3] * b[3],
                          a[0] * b[1] + a[1] * b[0] + a[2] * b[3] - a[3] * b[2],
                          a[0] * b[2] - a[1] * b[3] + a[2] * b[0] + a[3] * b[1],
                          a[0] * b[3] + a[1] * b[2] - a[2] * b[1] + a[3] * b[0] );
  }

  template <typename T>
  inline
  ostream& operator << ( ostream & os,  Quaternion<T> const & Q ) {
    Q.print(os);
    return os;
  }
}

#endif
```

### Quick search

### Table of Contents

- rang - Colors for your Terminal
- Zstr
- C++ API

«
hide menu

menu
sidebar
»

### Navigation

- index
- toc
- Utils »
- Program Listing for File Quaternion.hxx

© Copyright 2021, Enrico Bertolazzi.
Created using Sphinx 4.2.0.
